# Codon usage bias in yeasts and its correlation with gene expression, growth temperature, and protein structure

**DOI:** 10.3389/fmicb.2024.1414422

**Published:** 2024-07-08

**Authors:** Marcelo Baeza, Dionisia Sepulveda, Víctor Cifuentes, Jennifer Alcaíno

**Affiliations:** ^1^Departamento de Ciencias Ecológicas, Facultad de Ciencias, Universidad de Chile, Santiago, Chile; ^2^Facultad de Ciencias, Universidad de Chile, Santiago, Chile

**Keywords:** codon usage bias (CUB), relative synonymous codon usage (RSCU), preferred and non-preferred codons, codon clusters, environmental yeasts

## Abstract

Codon usage bias (CUB) has been described in viruses, prokaryotes, and eukaryotes and has been linked to several cellular and environmental factors, such as the organism's growth temperature, gene expression levels, and regulation of protein synthesis and folding. Most of the studies in this area have been conducted in bacteria and higher eukaryotes, in some cases with different results. In this study, a comparative analysis of CUB in yeasts isolated from cold and template environments was performed in order to evaluate the correlation of CUB with yeast optimal temperature of growth (OTG), gene expression levels, cellular function, and structure of encoded proteins. Among the main findings, highly expressed ORFs tend to have a more similar CUB within and between yeasts, and a direct correlation between codons ending in C and expression level was generally found. A low correspondence between CUB and OTG was observed, with an inverse correlation for some codons ending in C. The clustering of yeasts based on their CUB partially aligns with their OTG, being more consistent for yeasts with lower OTG. In most yeasts, the abundance of preferred codons was generally lower at the 5′ end of ORFs, higher in segments encoding beta strand, lower in segments encoding extracellular and transmembrane regions, and higher in “translation” and “energy metabolism” pathways, especially in highly expressed ORFs. Based on our findings, it is suggested that the abundance and distribution of preferred and non-preferred codons along mRNAs contribute to proper protein folding and functionality by regulating protein synthesis rates, becoming a more important factor under conditions that require faster protein synthesis in yeasts.

## Introduction

Codon usage bias (CUB) has been described in viruses, bacteria, fungi, plants, and animals (Hershberg and Petrov, 2008), and it has been proposed that it correlates with various biological factors such as the GC content, gene expression levels, tRNA abundance, gene translation initiation signals, and protein structure (Ikemura, 1985; Bulmer, 1991; Hooper and Berg, 2000; D'Onofrio et al., 2002; Chen et al., 2004; Hershberg and Petrov, 2008; Plotkin and Kudla, 2011; Parvathy et al., 2022). It has been proposed that microorganisms with a wide range of habitats tend to have a higher variation of CUB, which may help them to adapt efficiently to different conditions. On the other hand, microorganisms with similar phenotypic traits and that thrive in similar environments tend to have a similar CUB (Botzman and Margalit, 2011; Hart et al., 2018; Arella et al., 2021). These tendencies were observed in a metagenomic study of samples from different regions and substrates, including the sea, farm soils, human gut, and acid mine drainage (Roller et al., 2013). Microorganisms inhabiting cold regions have attracted considerable attention in recent decades due to their important ecological roles and potential in applied fields (Margesin, 2017), and it has been suggested the existence of a distinct CUB pattern for life in the cold. Comparison of coding sequences from psychrophiles revealed a decreasing trend for GC-rich and G/C-ending codons, while a preference for AGG (Ser) in thermophiles and CAA (Val) in mesophiles and psychrophiles (Khan and Patra, 2018). In a comparative genomic analysis of 78 *Cryobacterium* strains, a correlation between the CUB and growth temperature was reported. Strains having maximum growth temperature of 20°C or below tended to use synonymous codons ending in A or T, while those with a maximum growth temperature above 20°C preferred codons ending in G or C (Liu et al., 2020). A similar trend was found in a genomic study of the psychrophilic bacterium *Pseudoalteromonas shioyasakiensis* (Duan and Guo, 2021). A transcriptomic analysis of eight Antarctic yeasts revealed a variation in CUB associated with their growth temperatures: yeasts with growth temperatures below 20°C preferred codons AAC (Asn), GAG (Glu), CAC (His), ATC (Ile), AAG (Asn), and TTC (Phe), while those with growth temperatures above 20°C showed a CUB similar to that of mesophilic yeasts (Baeza et al., 2022). A preference for GGA (Gly) and CGA (Arg) has been described in the yeast *Mrakia psychrophila*, which grows optimally at 12–15°C (Su et al., 2016).

A pronounced CUB has been reported in highly but not in low expressed genes. Gene expression levels can be related to translation efficiency, and mutational pressure and natural selection have been proposed as the major forces driving CUB through selection of “translationally superior” codons (dos Reis et al., 2003; Goetz and Fuglsang, 2005; Klumpp et al., 2012; Novoa and Ribas de Pouplana, 2012; Frumkin et al., 2018; Liu, 2020; Iriarte et al., 2021). Furthermore, there is increasing evidence of the impact of CUB on gene expression and protein structure by influencing translation efficiency and accuracy, co-translational protein folding, and mRNA stability (Higgs and Ran, 2008; Shah and Gilchrist, 2010; Angov, 2011; Trotta, 2013; Liu, 2020). Stable mRNAs in *Saccharomyces cerevisiae*, such as those encoding enzymes involved in glycolysis and the large and small cytosolic ribosomal subunit proteins, tend to have a higher content of preferred codons (above 85% on average) and unstable mRNAs, such as those encoding polypeptides involved in yeast pheromone response and mitochondrial ribosomal proteins, have a lower content of preferred codons (an average of about 45%; Presnyak et al., 2015). Studies using cell-free translation systems from *Neurospora* and *Drosophila* showed that CUB affects the local translation elongation rates: optimal synonymous codons accelerate elongation, while non-optimal codons deaccelerate it, thereby affecting protein structure and function (Yu et al., 2015; Zhao et al., 2017). A genomic analysis of *Neurospora crassa, Escherichia coli, S. cerevisiae, Caenorhabditis elegans*, and *Drosophila melanogaster* showed that protein regions predicted to be unstructured are commonly encoded by non-preferred codons, while predicted structured regions are encoded by preferred ones (Zhou et al., 2015). However, it has been described that alpha-helices are mainly encoded by preferred codons and beta-strands by non-preferred codons in *E. coli* (Thanaraj and Argos, 1996).

In this work, a comprehensive comparative CUB analysis was conducted on 89 yeast strains from different species isolated from diverse environments to evaluate its correlations with several parameters, including gene expression levels, growth temperatures, GC composition, response to cold stress, and some predicted protein properties.

## Methods

### Yeast culture conditions

*Phaffia rhodozyma* strains were cultivated in YM or minimal medium supplemented with 2% glucose or maltose and incubated at 10, 15, 22, or 26°C with 150 r.p.m. orbital shaking. Growth was monitored by the optical density of the culture at 600 nm, and growth rates were calculated from the exponential growth phase. The temperature at which the yeast strain had the highest growth rate was considered optimal. The four *P. rhodozyma* isolates showed higher growth rates between 10 and 15°C, and the optimal growth temperature was 15°C for isolates UCD 647-385, Av, and VOH, and 10°C for CBS 6938 (Supplementary Table 1). Data on optimal temperature for growth for other yeasts were obtained from previous works (Baeza et al., 2022) and databases such as American Type Culture Collection (ATCC), Westerdijk Institute, Institute for Systems Biology, National Collection of Yeast Cultures.

### RNA-seq

*P. rhodozyma* isolates were grown until the stationary phase in 100 ml of different media at 22°C, as indicated in Supplementary Table 2. Cultures were centrifuged at 4,000 *g* for 5 min, and total RNA was purified using TRIzol (Invitrogen). The RNA samples having 260/280 and 260/230 >1.9 were stored in 50% ethanol with 0.3 M sodium acetate and sent to Macrogen Inc. for next-generation sequencing using the protocols and platforms indicated in Supplementary Table 2. RNA integrity (RIN) was determined at Macrogen Inc., and samples with RIN > 7 were processed. Eleven transcriptomes were determined for the *P. rhodozyma* isolates in this work, eight for strain UCD 67-385, and three for the Chilean isolates VOH and AVHN2 (Loto et al., 2012). The raw data statistics are indicated in Supplementary Table 2 and are available at NCBI, BioProject accession number PRJNA966916. The transcriptomes of the Antarctic yeast isolates were previously described (Baeza et al., 2022), and the Sequence Read Archive (SRA) of the transcriptomes of other yeast species (a total of 1,416 SRA files) were downloaded from the NCBI database. The abbreviations, growth temperature, and codes of the SRA files of yeast species used in this work are listed in Supplementary Table 3.

### Bioinformatic analysis

Contigs of at least 210 nt were assembled from each SRA using Tadpole assembler 38.84 (plugin in Geneious prime v11), and the expression levels of the ORFs in each yeast were determined by mapping each SRA (RNAseq plugin in Geneious prime v11) and expressed in reads per kilobase per million mapped reads (RPKM; Mortazavi et al., 2008). The maximum RPKM value was considered for contigs in yeast with multiple transcriptome data, and only contigs with RPKM values ≥ 10 were included in the subsequent analysis. The open reading frames (ORF) ≥ 210 nt in each contig were predicted, translated *in silico*, and compared to KAAS—KEGG Automatic Annotation Server (Moriya et al., 2007), using the default parameters of GHOSTX (Suzuki et al., 2014) and gene datasets for eukaryotes, and only annotated ORFs were considered for further analysis. The secondary structures (alpha-helices, beta-sheets, turns, and coils) and subcellular localization (cytoplasmic, transmembrane, and extracellular) were predicted from translated ORFs using the EMBOSS garnier plugin in Geneious prime v11 (Rice et al., 2000; Viklund and Elofsson, 2004).

### Relative synonymous codon usage calculation and comparative analysis

The calculation of RSCU was performed for each ORF in each yeast as the ratio between the frequency of a codon and the expected frequency if all synonymous codons were used equally. An RSCU = 1 indicates that the codon usage pattern has no preference, while an RSCU > 1 indicates that the codon has a preference. Codons with RSCU ≥ 1.5 are classified as high-frequency codons, and those found in the highest expressed ORFs (RPKM > 1,000) were referred to as “preferred codons” in this work. Normality of the data was tested using Llliefors, Kolmogorov_Smirnov, Anderson Darling, and DÁgostino-K squared, all of which rejected normality; therefore, comparative analyses were performed using the non-parametric Kruskal-Wallis test and Dunn's multiple comparisons. Comparative analyses were performed for each codon between ORFs grouped by parameter when appropriate. Five groups were classified by expression level (L1, 10 ≥ RPKM ≤ 100; L2, 100 < RPKM ≤ 300; L3, 300 < RPKM ≤ 500; L4, 50 < RPKM ≤ 1,000; and L5, 1,000 < RPKM), eight groups according to % GC (gc1, 10 ≥ %GC ≤ 20; gc2, 20 < %GC ≤ 40; gc3, 40 < %GC ≤ 60; gc4, 60 < %GC ≤ 80), and 22 groups according to predicted cellular pathways as indicated in Table 1.

**Table 1:** List of abbreviations used in this work for cellular pathways.

**Cellular pathway**	**Abbreviation**
Amino acid metabolism	Aam
Biosynthesis of other secondary metabolites	Boosm
Carbohydrate metabolism	Cmet
Cell growth and death	Cgad
Cell motility	Cmom
Cellular community—eukaryotes	Cc-e
Energy metabolism	Em
Folding, sorting, and degradation	Fsad
Glycan biosynthesis and metabolism	Gbam
Lipid metabolism	Lm
Membrane transport	Mt
Metabolism of cofactors and vitamins	Mocav
Metabolism of other amino acids	Mooaa
Metabolism of terpenoids and polyketides	Motap
Nucleotide metabolism	Nm
Replication and repair	Rar
Signal transduction	St
Signaling molecules and interaction	Smai
Transcription	Transc
Translation	Transl
Transport and catabolism	Tac
Xenobiotics biodegradation and metabolism	Xbam

## Results

### Codon bias in yeasts and correlation with growth temperature, expression levels, and %GC

**Figure 1:**
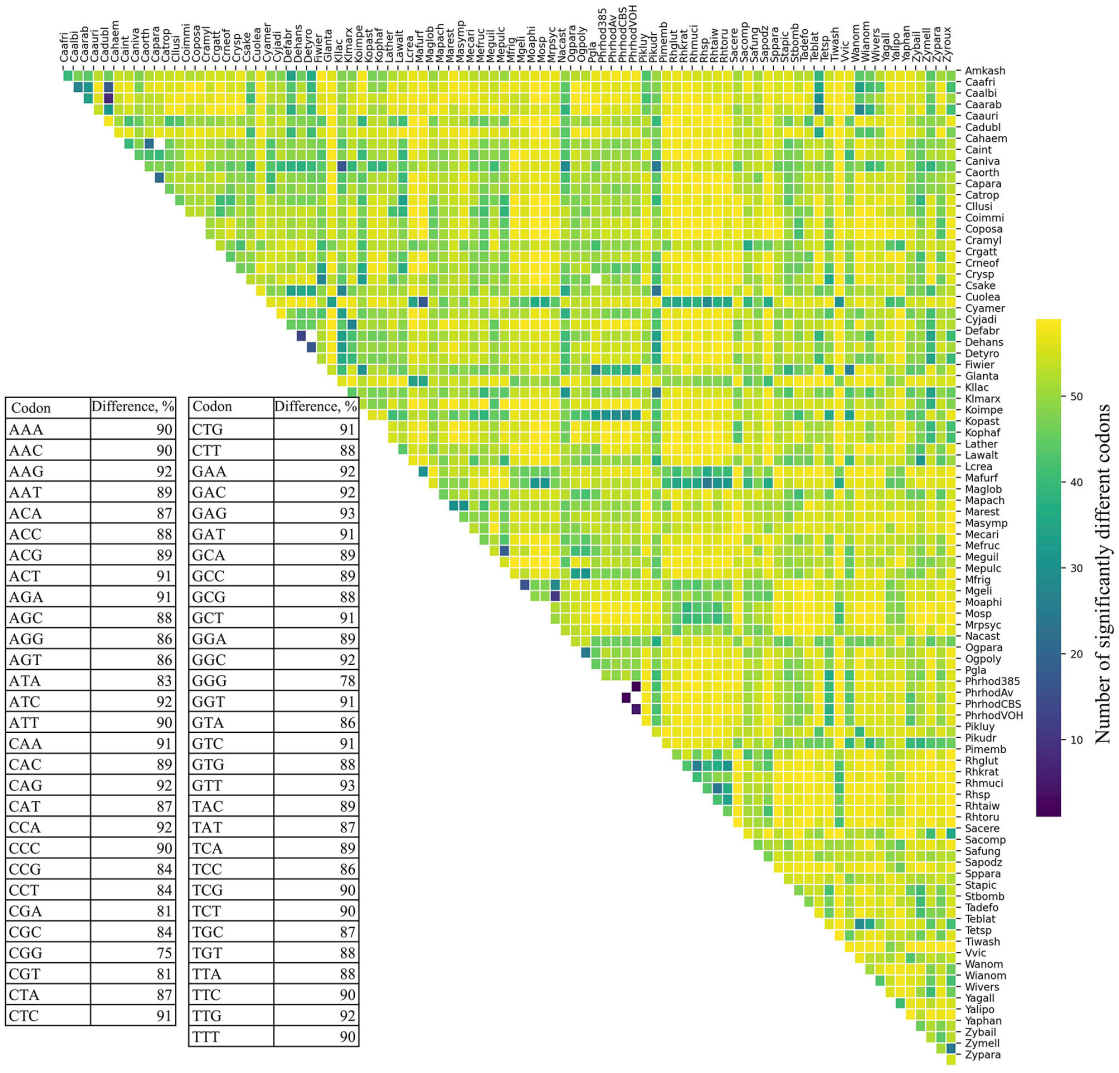
Variation in synonymous codon usage among yeasts. The RSCU values of each codon in all ORFs were compared between different pairs of yeasts. The table shows the percentage of significant differences (*p* ≤ 0.05) for each codon in all comparisons. The number of significantly different codons between yeast pairs is shown in the heatmap, and their distribution is in the box plot. The full names of the species are listed in the Supplementary Table 3.

RSCU was calculated for each ORF, and codon values were compared across yeasts. All codons showed a high percentage of significant difference in RSCU values between yeast pairs, ranging from 75% (CGG) to 93% (GAG and GTT; table in Figure 1). The number of significantly different codons between yeast pairs varied from eight codons in *C. albicans* vs. *C. dubliniensis* to 59 codons in 258 yeast pairs, such as *Candida auris* vs. *Moesziomyces aphidis, Candida haemuloni* vs. *Rhodotorula toruloides*, and *Candida orthopsilosis* vs. *Tilletiopsis washingtonensis* (heatmap in Figure 1). The median number of different codons was 54, with an interquartile range of 8 (boxplot in Figure 1).

**Figure 2:**
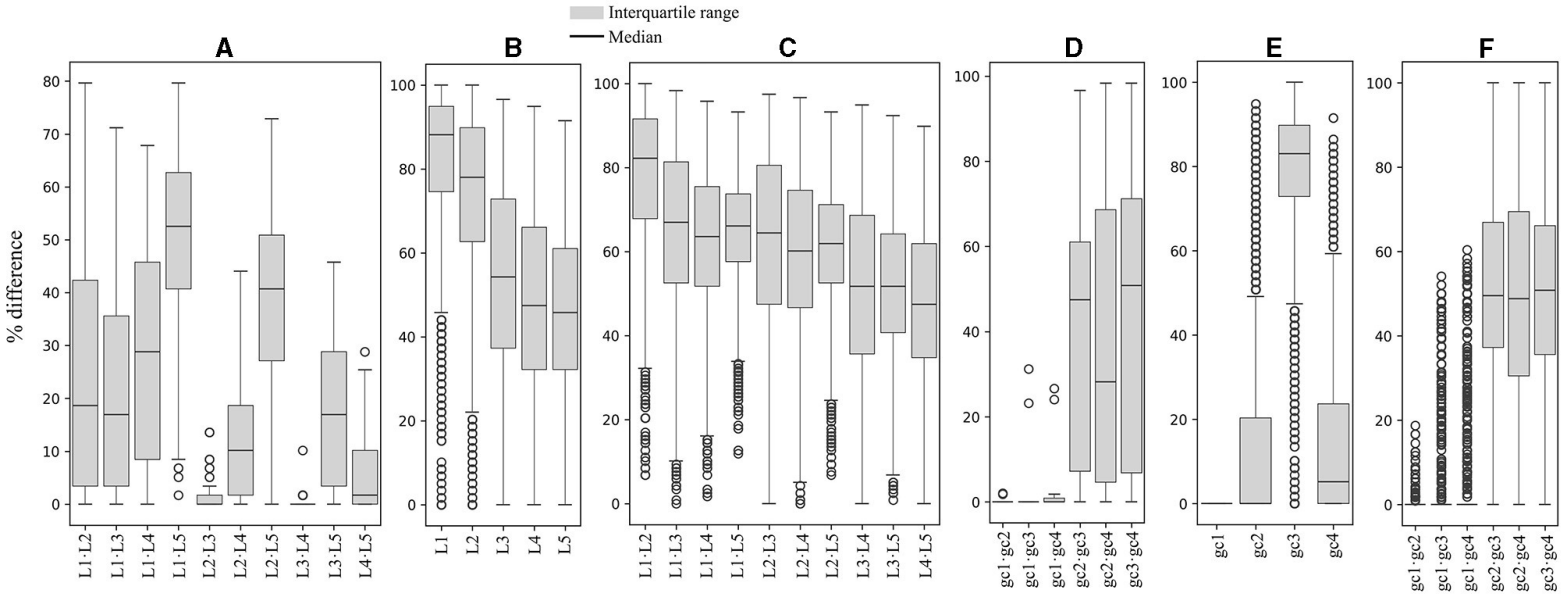
Variation in synonymous codon usage within and across yeasts considering the ORF expression level and %GC. The RSCU of each codon was compared between ORFs grouped by their expression level and %GC within and between yeasts. Median and distribution of significant differences (*p* ≤ 0.05) between expression level groups within **(A)** and between **(B, C)** yeasts. Median and distribution of significant differences (*p* ≤ 0.05) between %GC groups within **(D)** and between **(E, F)** yeasts. L1–L5 or gc1–gc4 correspond to the groups of ORFs classified by expression level or %GC from lowest to highest.

The RSCU values for each codon were compared between ORFs grouped by expression level and by %GC within and between yeasts. As shown in Figure 2, significant differences in RSCU between groups increase on average as their difference in expression level increases, being maximum between groups of ORFs with RPKM values between 10 and 100 (L1) and those with RPKM higher than 1,000 (L5; Figure 2A). Regarding %GC, minor RSCU differences were found between the group with 1–20 %GC (gc1) and the others, probably due to the low number of yeasts (15) and ORFs (21–542) in this category. Among the other groups, the average difference was variable in all yeasts analyzed, with the highest differences between those with 41–60 %GC (gc3) and 61–80 %GC (gc4), and the lowest between those with 21–40 %GC (gc2) and 61–80 %GC (gc4; Figure 2D). When comparing RSCU values between yeasts, higher similarities were found between highly expressed groups, whether comparing the same or different groups (Figures 2B, C). Regarding the %GC, the 41–60 %GC group showed the highest difference across yeasts. When comparing different %GC groups, the observed differences were similar, ranging from 48–50 %GC, except for the 1–20 %GC group (Figures 2E, F). These results suggest that, between the two parameters analyzed, codon usage appears to be more significantly influenced by the expression level of genes than by their GC content.

**Figure 3:**
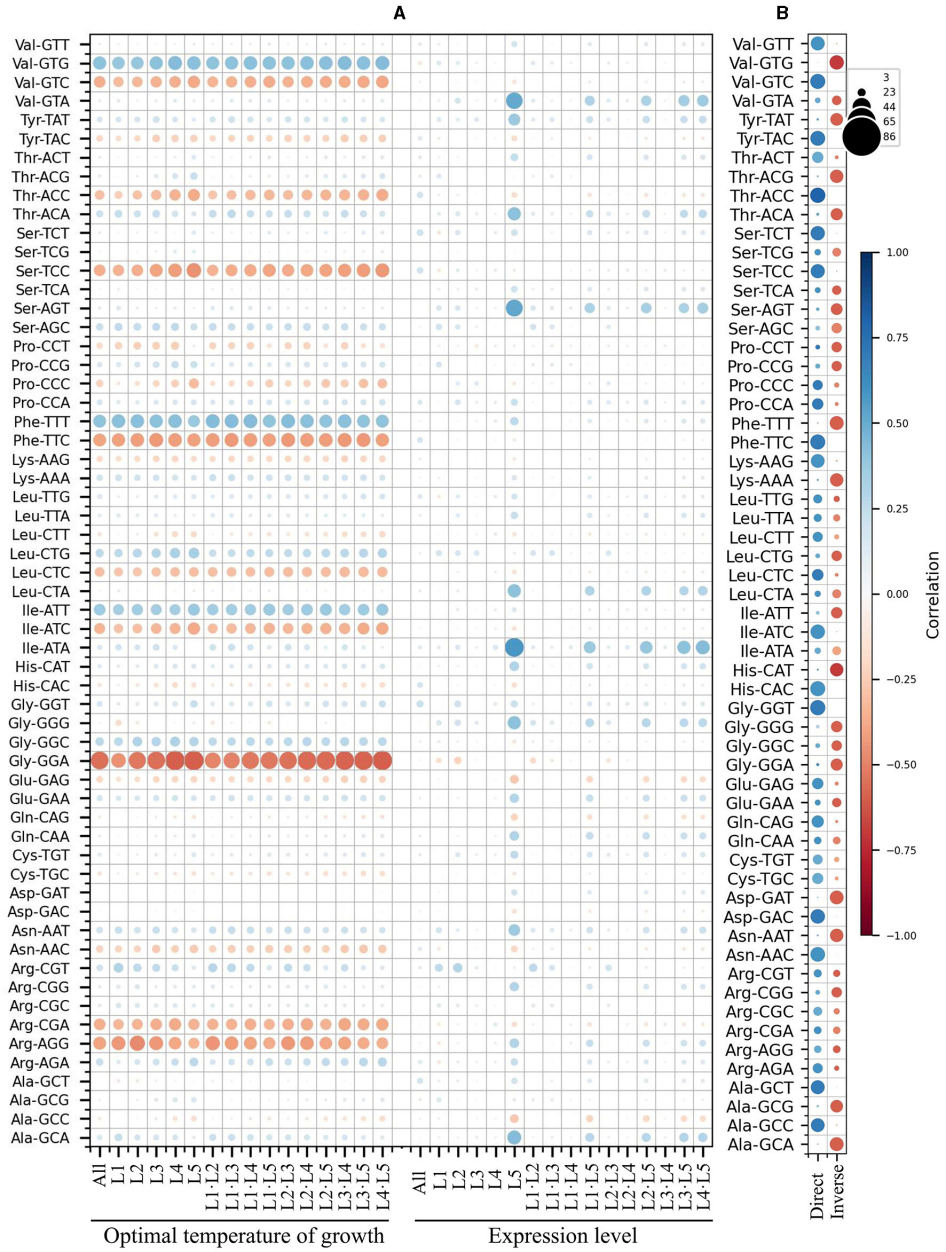
Correlation between synonymous codon usage and the yeast optimal temperature of growth or ORF expression level. A matrix was created containing all combinations of yeast strains and expression level groups for each codon. The differences in the corresponding values of RSCU, optimal temperature of growth, and expression level were calculated. These differences were used for principal component analysis (PCA) between different yeast strains **(A)** and within each yeast strain **(B)**, as well as between the same or different expression level groups. In **(A)**, the color and size of the circle represent the correlation values. In **(B)**, the circle color corresponds to the correlation value, and its size represents the number of yeasts with the corresponding correlation. L1–L5 correspond to the ORF expression level groups from lowest to highest.

Since the analysis based on the expression level groups gave more consistent results, this parameter was selected for further correlation analyses, including the yeast optimal temperature of growth (OTG). In the analyses of correlations between RSCU and OTG, considering only correlation values ≥ |0.4|, inverse correlations were found for the codons GGA (Gly), AGG (Arg), TTC (Phe), TCC (Ser), CGA (Arg), and GTC (Val), and direct correlations for the codons ATT (Ile), TTT (Phe), and GTG (Val) when considering the whole data set (Figure 3A). The same tendency was observed in analyses of OTG values grouped by expression level group, comparing the same or different expression level groups, with additional inverse correlations for ATC (Ile) and ACC (Thr). Regarding correlations between RSCU and expression level, no correlations were observed when considering the entire data set. However, when different expression level groups were compared, direct correlations for GGG (Gly), ATA (Ile), AGT (Ser), CTA (Leu), GTA (Val), GCA (Ala), TAT (Tyr), ACA (Thr), and AAT (Asn) were observed in the highly expressed groups.

When comparing the expression level groups in each yeast, more codons having correlations to expression level were identified (Figure 3B). Considering only correlations ≥ |0.5| found in most yeasts (in at least 60 of them), direct correlations were detected for ACC (Thr), GGT (Gly), GTC (Val), TTC (Phe), CAC (His), TAC (Tyr), AAC (Asn), GAC (Asp), ATC (Ile), TCT (Ser), TCC (Ser), GCT (Ala), GTT (Val), GCC (Ala), AAG (Lys), CAG (Gln), CTC (Leu), CCA (Pro), GAG (Glu), ACT (Thr), and TGC (Cys). Inverse correlations were found for GTG (Val), TTT (Phe), GCA (Ala), GAT (Asp), CAT (His), ACG (Thr), AAT (Asn), AAA (Lys), GCG (Ala), TAT (Tyr), GGA (Gly), ACA (Thr), AGT (Ser), GGG (Gly), ATT (Ile), GGC (Gly), CGG (Arg), CCT (Pro), CTG (Leu), and AGC (Ser). These results suggest some correlation between codon usage and OTG in yeast, but it was less pronounced than expression level, both in terms of codon numbers and correlation values. Furthermore, the correlation between codon usage and expression level was more noticeable when comparisons were made between expression level groups within each yeast.

### Similarity in codon usage among different yeasts

**Figure 4:**
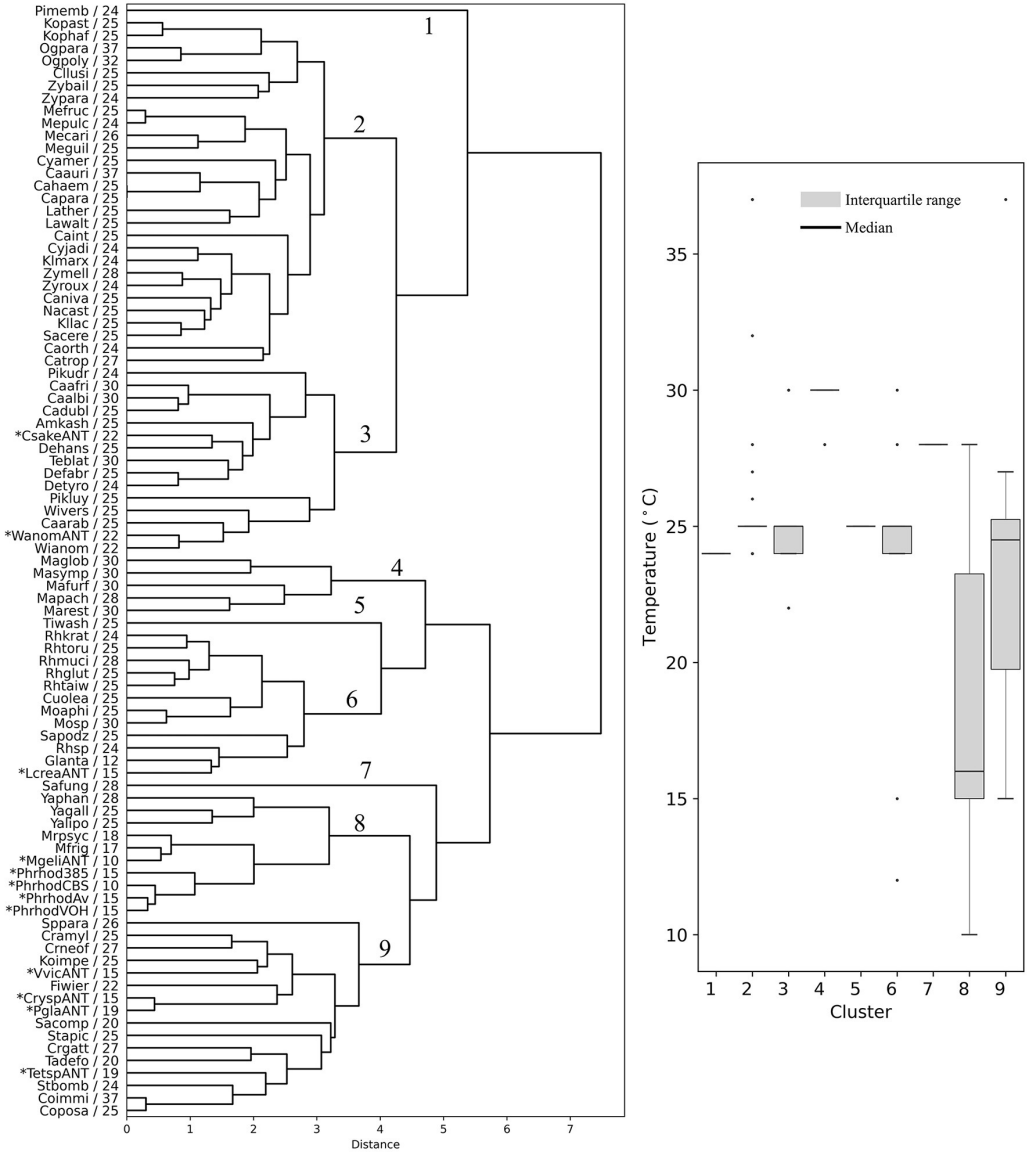
Hierarchical clustering of yeasts based on codon usage. Dendrograms were constructed considering the RSCU of codons calculated from the highest expression level group of ORFs. The yeast abbreviation and optimal temperature of growth are indicated in the dendrograms (the full names of the species are given in the Supplementary Table 3). Asterisks indicate yeasts isolated from Antarctica and *P. rhodozyma* isolates for which the optimal temperature of growth was determined in this work. The box plot shows the distribution of the yeasts' optimal temperature of growth in each hierarchical group (1–9).

The yeasts were hierarchically clustered according to their codon usage calculated from ORFs from the highest expressed group (L5, RPKM >1,000, Supplementary Table 4), resulting in a dendrogram with nine main groups (Figure 4). There were five groups that contained at least 10 members from different yeast genera that showed a similar OTG (Figure 4, boxplot). One group, group 6, had only species of the genus *Malassezia* with an OTG of 28–30°C. Group 2 was the most numerous with 28 members, the majority with OTG between 24 and 25°C. Antarctic yeasts with OTG of 22°C (*Candida sake* and *Wickerhamomyces anomalus*) grouped with other 13 yeasts with OTG from 22 to 30°C (group 3), while the Antarctic yeasts with OTG of 15 and 19°C grouped with other 12 yeasts with OTG mainly from 20 to 27°C. Antarctic yeast isolates *M. gelida* and *P. rhodozyma*, with the lowest OTG values determined by our group, grouped with other cold-adapted *Mrakia* species, and with *Yarrowia* species, corresponding to the group with the lowest median for OTG (16°C) of all groups. It can be observed that mesophilic yeasts (OTG ≥ 20°C) generally group in ways that cannot be attributed only to their OTG. On the other hand, although seven yeasts with the lowest OTG were grouped with two mesophilic yeasts in group 8, they formed a separate subgroup, suggesting a more similar codon usage among psychrophilic yeasts.

### Preferred codon content in cellular pathways and regulated ORFs

**Figure 5:**
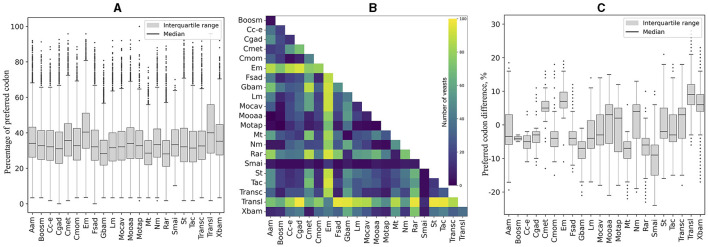
Comparison of preferred codon content in cellular pathways. The content of preferred codons was calculated in each ORF classified according to its predicted cellular pathway and compared across yeasts. **(A)** Distribution of the percentage of preferred codons considering all ORFs. **(B)** Number of yeasts with significant differences between the compared cellular pathways. **(C)** Distribution of significant differences in the content of preferred codons between a pathway and the others. The full names of the cellular pathways are listed Table 1.

The codons with an RSCU ≥ 1.5 calculated from highly expressed ORFs (L5, RPKM >1,000, Supplementary Table 4) were considered “preferred” in this work. The content of preferred codons was calculated in each ORF, grouped according to the cellular pathways in which the encoded protein was predicted to be involved, and compared within each yeast. As shown in Supplementary Figure 1 and summarized in Figure 5A, the pathways with the highest percentage of preferred codons in most yeasts were “translation,” ranging from 32% in *Kockovaella imperatae* to 58% in *Naumovozyma castellii* (a median of 40%), and “energy metabolism,” ranging from 30% in *Coccidioides posadasii* to 56% in *Tilletiopsis washingtonensis* (a median of 41%). When each pathway was compared with the others, “translation” and “energy metabolism” were significantly different in the largest number of yeasts (Figure 5B). In addition, when estimating the difference in the percentage of preferred codons between each pathway vs. the others with significant differences, these two pathways showed the highest percentage of preferred codons (Figure 5C).

**Figure 6:**
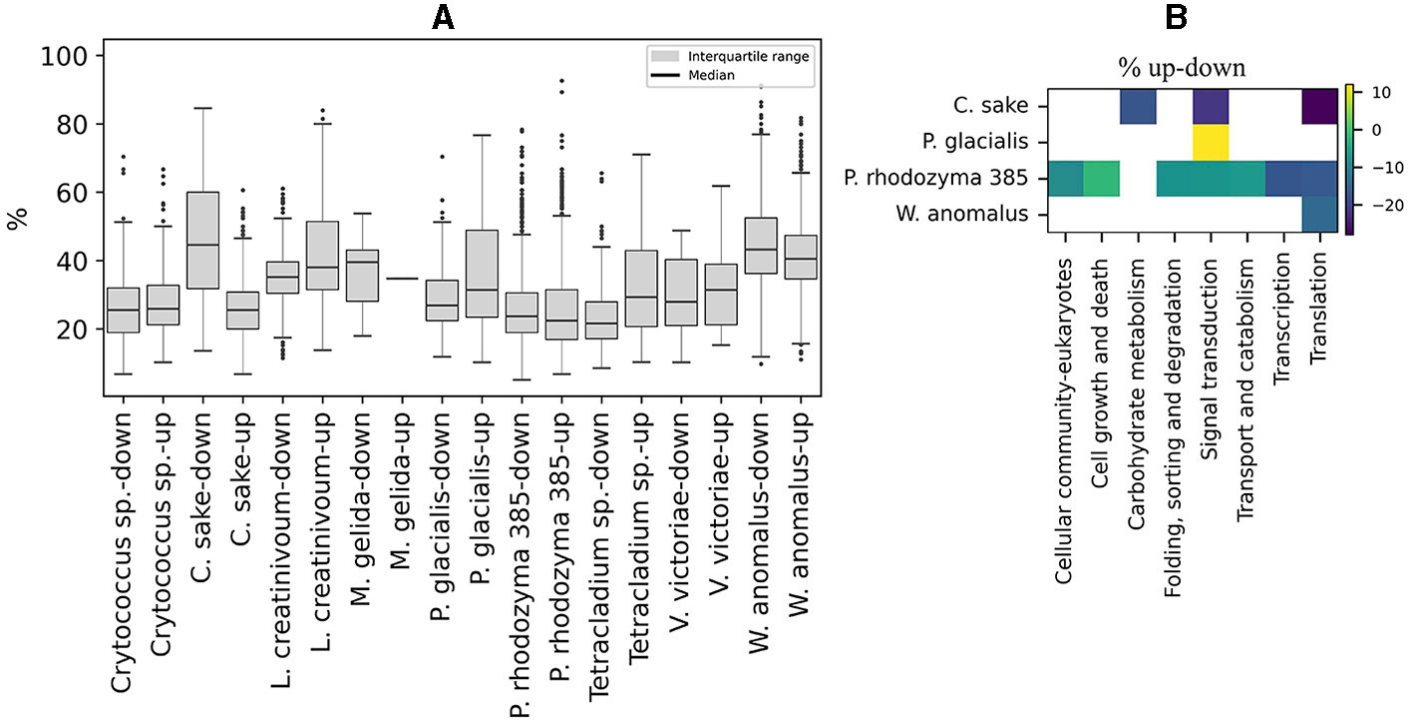
Codon preference content in regulated ORFs. **(A)** Percentage of preferred codons in upregulated (log2 ≥ 1) and downregulated (log2 ≤ −1) ORFs after cold stress in Antarctic yeasts and after growth in two different carbon sources (glucose or maltose), in *P. rhodozyma* 385 isolates. **(B)** Difference in the percentage of up- minus down-regulated (% up-down) ORFs in cellular pathways that are significantly different in each yeast.

The content of preferred codons was also analyzed in ORFs that were up- or down-regulated in Antarctic yeasts subjected to cold stress and in *P. rhodozyma* UCD 67-385 cultivated on different carbon sources (glucose or maltose). As shown in Figure 6A, the distribution of the percentage of preferred codons and the median in up- and down-regulated ORFs was similar in most of the yeasts analyzed, except for *C. sake* in which down-regulated ORFs showed a higher percentage of preferred codons than up-regulated ORFs. When comparing the preferred codon content between up- and down-regulated ORFs classified by cellular pathways, significant differences were detected only in a few pathways (from one to seven) in *C. sake, P. glacialis, P. rhodozyma* UCD 67-385, and *W. anomalus*, with a generally lower percentage of preferred codons in up-regulated ORFs than in down-regulated ones, except for “signal transduction” in *P. glacialis* (Figure 6B). Therefore, in general, there is no difference in the content of preferred codons between up- and down-regulated ORFs, at least under the conditions analyzed here.

### Distribution of preferred codons in ORFs and structural properties of coded proteins

**Figure 7:**
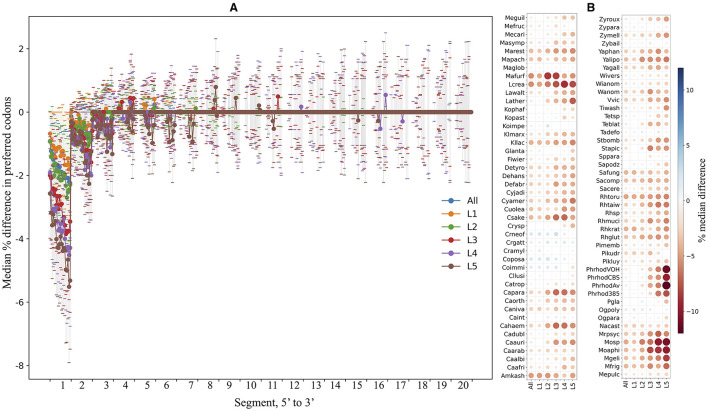
Preferred codon content analysis along ORFs. The yeast ORFs were fragmented *in silico* into non-overlapping segments of 30 nt, and the percentage of preferred codons in each segment was calculated. **(A)** The median differences in preferred codon content between a segment and the contiguous downstream segments for the first 20 5′-end segments of the ORFs. **(B)** The median differences (color of circles; sizes of circles represent absolute values of median) in preferred codon content between the first 5′-end segment and the 20 downstream segments in each yeast. Analyses were performed using the entire data set (“All”) or subsets of ORFs classified by expression level (“L1–L5”). L1–L5 correspond to the ORF expression level group from lowest to highest. See Supplementary Table 3 for the full names of yeasts.

The ORFs were fragmented *in silico* into non-overlapping segments of 30 nt, and the percentage of preferred codons within each segment was calculated and compared in each yeast. Figure 7A shows the median differences in the percentage of preferred codons between a single segment and the contiguous 20 downstream segments for the first 5′ end 20 segments of the ORFs from all yeasts. An evident lower content of preferred codons was observed when comparing the first segments to the others, a tendency that gradually decreased for the downstream segments and was more pronounced in ORFs with higher expression levels. Figure 7B shows the median difference in the percentage of preferred codons between the first 5′ segment of the ORFs and the subsequent 20 segments in each yeast. In most yeasts, the first segments of the ORFs had a lower percentage of preferred codons, which was more noticeable in the groups of ORFs with higher expression levels. This pattern was particularly pronounced in the *P. rhodozyma* isolates, *Moesziomyces* sp., *Moesziomyces aphidis, M. gelida, L. creatinivorum, Lachancea thermotolerans*, and *M. frigida*. These results showed a general tendency for ORFs in the yeasts analyzed here to have a lower content of preferred codons at the 5′ end, more pronounced in highly expressed ORFs, which would imply a slower translation start, as discussed below.

**Figure 8:**
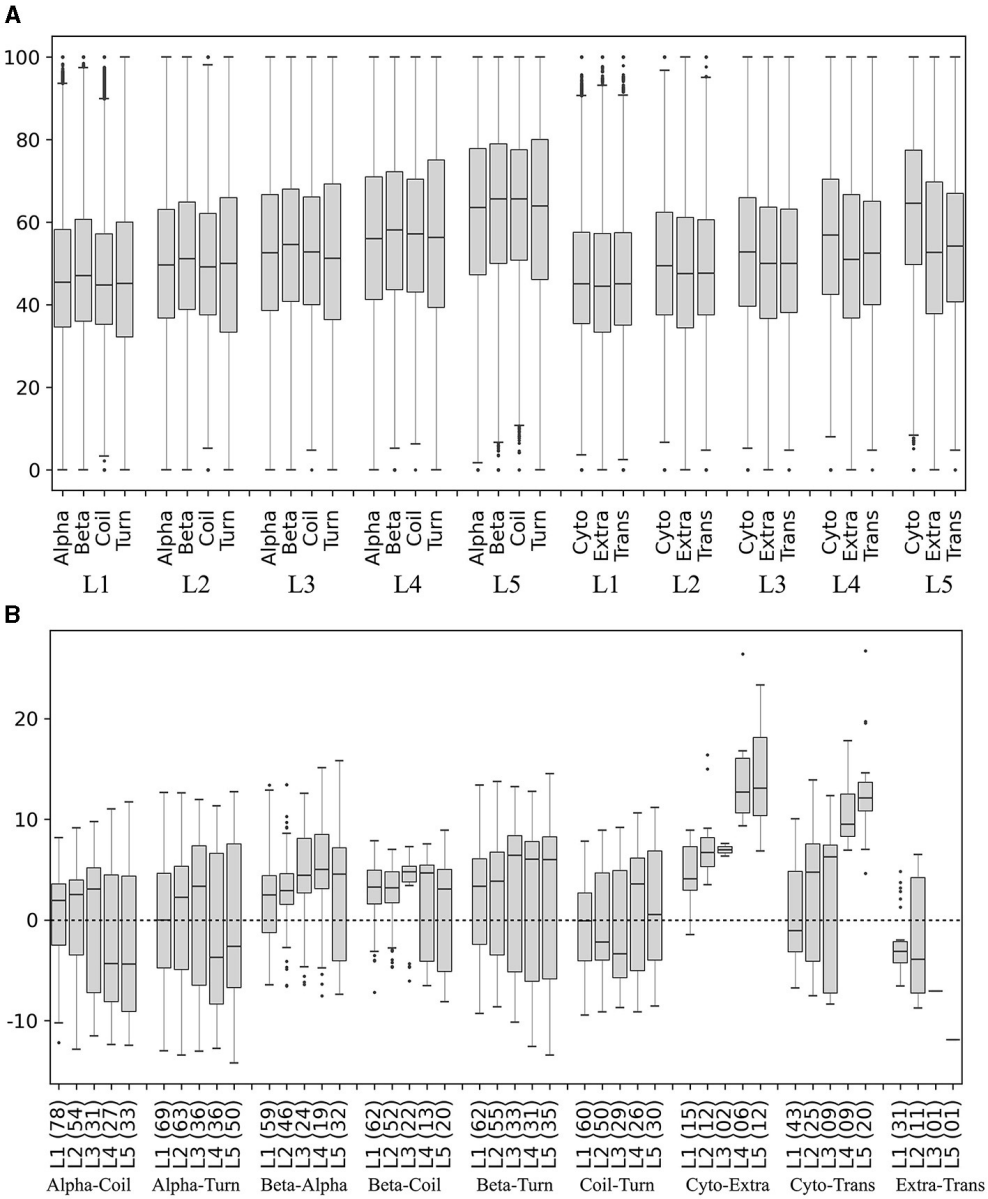
Preferred codon content in ORF segments encoding secondary structures and subcellular localizations. The secondary structures and subcellular localizations were predicted in the translated ORFs, and the percentage of preferred codons in the corresponding ORF segments was calculated in each yeast. **(A)** Distribution of preferred codon percentage in each category in each ORF expression level group, considering data from all yeasts. **(B)** Difference of preferred codon percentages between categories in each ORF expression level group in each yeast. Only the distribution of the percentage differences between significantly different categories (*p* ≤ 0.05) is shown in the box plot, and the number of yeasts in which significant differences were found is given in parentheses. Alpha, alpha helix; Beta, beta strand; Cyto, cytoplasmic; Trans, transmembrane; Extra, extracellular. L1–L5 correspond to the ORF expression level group from lowest to highest.

The secondary structures (beta-strands, alpha-helices, turns, and coils) and subcellular localizations (cytoplasmic, extracellular, and transmembrane) in the translated ORFs were predicted, and the percentage of preferred codons in the corresponding coding segments was calculated and compared in each yeast. The percentage of preferred codons in all secondary structures increased as the expression level of the ORF group increased. In all expression level groups, the beta strand showed a higher percentage of preferred codons than the other secondary structures (Figure 8A), and no clear trend was observed between the alpha helix with respect to coil and turn. The percentage of preferred codons in subcellular localizations was lower in extracellular and transmembrane than in cytoplasmic, a tendency that was more pronounced in ORFs with higher expression levels (Figure 8A). When comparing the categories in each yeast, the beta strand does indeed show a higher percentage of preferred codons than the other secondary structures at all expression levels; however, the number of yeasts that showed significant differences in these comparisons tended to decrease as the expression level of the group increased (Figure 8B). In the case of subcellular localizations, the cytoplasmic showed a higher content of preferred codons than the other two, especially in the groups of ORFs with higher expression levels (L4 and L5). However, only a small fraction of the analyzed yeasts (between 7 and 22%) were the ones that showed significant differences in these comparisons (Figure 8B). Thus, the higher content of the preferred codon in predicted beta strands and cytoplasmic proteins was the more consistent trend.

## Discussion

In this work, the use of synonymous codons in different yeasts was analyzed in relation to various factors including their temperature of growth, gene expression levels, protein structure and subcellular localization, and cellular pathways where proteins would be involved. Our results showed that highly expressed ORFs tend to have a more similar RSCU in both, within a yeast and among different yeasts. These findings are consistent with the significant bias observed in highly expressed genes, for which it has been suggested that selective pressures would favor certain codons to optimize the efficiency and speed of protein translation (dos Reis et al., 2003; Goetz and Fuglsang, 2005; Klumpp et al., 2012; Novoa and Ribas de Pouplana, 2012; Frumkin et al., 2018; Liu, 2020; Iriarte et al., 2021). When analyzing the relationship between RSCU and yeast OTG, correlations (ranging from |0.4| to |0.6|) were found for 11 codons, eight inverse and three direct. Five of 16 codons ending in C showed an inverse correlation, two of 14 codons ending in A showed an inverse correlation, two of 16 codons ending in T showed a direct correlation, and of the 13 codons ending in G, one had an inverse correlation, and one had a direct correlation. These findings are not consistent with the A/T preference at the third codon position reported in prokaryotes growing at low temperatures (Liu et al., 2020; Duan and Guo, 2021). However, other work suggested that CUB could not be attributed to a selective pressure related to the microorganism temperature for growth as an unusual clustering based on CUB of an archaeal psychrophile with thermophiles and hyperthermophiles was obtained (Lobry and Necşulea, 2006). Regarding ORF expression levels, it has been reported that highly expressed genes with low translation rates are severely depleted in fast growing cells (Hausser et al., 2019), which aligns with the hypothesis that highly expressed genes would require a rapid translation elongation to minimize ribosome sequestration and alleviate ribosome shortage (Yang et al., 2014). In the yeasts studied in this work, stronger correlations (between |0.5| and |0.8|) were observed between CUB and ORF expression levels. These correlations were generally direct for C, especially for codons varying only between C and T, and both inverse and direct for G, being direct when the only possibility of variation was between G and A. These findings do not align with results described in *S. cerevisiae*, where genes with AT-rich codons had a faster translation than GC-rich ones (Gardin et al., 2014).

CUB has also been related to cellular fitness, the lifestyle of microorganisms, and their need to adapt efficiently to different environments (Botzman and Margalit, 2011; Roller et al., 2013; Hart et al., 2018; Arella et al., 2021). The yeasts selected for this study were those for which transcriptomic and growth temperature data were available, either in databases or determined by us, resulting in a collection of yeasts isolated from different locations and substrates. For Antarctic yeasts and *P. rhodozyma* isolates, the clustering of yeasts based on their CUB was generally consistent with OTG, as those with higher OTG (22°C) grouped with yeasts with OTG around 25°C, and those with lower OTG (19°C or below) clustered with other cold-adapted yeasts with OTG below 25°C. However, this consistency was not observed in the clusters of most of the other yeasts analyzed. In the case of Antarctic yeasts and *P. rhodozyma* isolates, their OTG is consistent with their growth rates (Baeza et al., 2021; Supplementary Table 1); however, the growth rate is unknown for most of the yeasts included in this work and could be one of the factors influencing clustering, as yeasts with similar OTG may not have similar growth rates.

The number of preferred codons (with RSCU ≥ 1.5 in highly expressed ORFs) varied among yeasts from 12 to 25 (19 on average), and codons such as ACC (Thr), TAC (Tyr), ATC (Ile), AAG (Lys), TCC (Ser), and AAC (Asn) were preferred by most yeasts. Among the predicted cellular pathways, “translation” and “energy metabolism” were the ones whose associated ORFs had the highest content of preferred codons in most yeasts. This is an interesting result as it has been suggested that codon usage bias in bacteria is likely to initially evolve in genes related to the translation machinery rather than in other cellular functions, thereby improving the efficiency of translation machinery, which in turn significantly boosts the translation efficiency of other genes (González-Serrano et al., 2022).

In transcriptomic studies of organisms such as *S. cerevisiae, Schizosaccharomyces pombe*, and *C. elegans* subjected to various stress conditions, an overrepresentation of codons read by rare tRNAs was observed (Gingold et al., 2012). In the analysis of Antarctic yeasts subjected to cold stress and of *P. rhodozyma* UCD 67-385 cultured on different carbon sources in this work, the percentage of preferred codons was similar in up and down regulated ORFs. Although significant differences were found in the content of preferred codons between up and down regulated ORFs classified by metabolic pathways, generally lower in up regulated ORFs, differences were found only in four yeasts in a few pathways in each. These variable results are expected, as the stress response in yeasts involves complex and extensive mechanisms, including a global reduction of the cellular metabolism, activation of specific stress response genes, and translational adaptation requiring a coordinated balance between the tRNA epitranscriptome and codon bias (Chan et al., 2018).

CUB has been implicated in the regulation of gene expression and protein structure by influencing the translation efficiency and accuracy, cotranslational protein folding, and mRNA stability (Higgs and Ran, 2008; Shah and Gilchrist, 2010; Angov, 2011; Trotta, 2013; Liu, 2020). The presence of “rare” codons at the 5′ end of mRNAs has been proposed to create a “slow ramp” effect that reduces ribosome collisions along the coding sequence, thereby preventing the detrimental activation of ribosome quality control mechanisms dependent on colliding ribosomes (Tuller et al., 2010; Verma et al., 2019). A general trend toward a lower content of preferred codons at the 5′ end of ORFs was observed in the yeasts analyzed in this study, which was more pronounced in highly expressed than in low expressed ORFs, supporting the “slow ramp” hypothesis and this effect would be more relevant in genes under faster translation. Other aspects that could be related to the content and distribution of preferred codons along mRNAs include segments encoding structural or functional regions of proteins or their subcellular localization. It has been reported in *N. crassa, E. coli, S. cerevisiae*, C. *elegans*, and *Drosophila melanogaster* that protein regions predicted to be structured are mainly encoded by optimal codons, whereas unstructured regions are encoded by non-optimal ones (Zhou et al., 2015). However, in another study in *E. coli* it was found that alpha helices are encoded mainly by optimal codons, while beta strands and coils by non-optimal codons (Thanaraj and Argos, 1996). In addition, it has been reported in *E. coli* and some yeasts that ORFs encoding membrane-associated proteins involved in targeting, insertion, or interaction with other proteins, as well as ORF regions encoding secondary structures of polypeptides, tend to be rich in rare codon clusters (Zhang et al., 2009; Chartier et al., 2012; Pechmann and Frydman, 2013; Fluman et al., 2014). In most of the yeasts analyzed in this work, the ORF segments encoding beta strands had a higher percentage of preferred codons than those encoding alpha helices, coil, or turn, and those encoding transmembrane protein segments or extracellular proteins had a lower percentage than cytoplasmic. Several lines of evidence support the existence of a balance between optimal and non-optimal codons in mRNAs, including the presence of codon clusters that would contribute to the regulation of global and local rates of protein synthesis. It has been hypothesized that this balance would help to generate translation pauses to ensure proper protein folding, availability of free ribosomes for highly expressed ORFs under rapid growth conditions, regulation of translation initiation, and proper protein targeting (Chaney and Clark, 2015; González-Serrano et al., 2022; Parvathy et al., 2022). For example, the cross-pathway control protein 1 (CPC-1) gene from *N. crassa*, has a non-optimal RSCU profile rich in NNU codons, which was suggested to be important for maintaining a proper protein structure and function (Lyu and Liu, 2020). Supporting this idea, studies using cell-free translation systems from *Neurospora* and *Drosophila* showed that while optimal synonymous codons can enhance the translation elongation rate, non-optimal codons can decelerate it, with consequential impacts on the structure and function of proteins (Yu et al., 2015; Zhao et al., 2017).

Finally, assuming that highly expressed ORFs tend to be translated faster, the findings from this work strongly suggest a relationship between CUB and the rate of protein synthesis, which in turn is related to cellular growth rate and gene expression levels in yeasts. Regulation of protein synthesis rates contributes to proper protein folding and subcellular targeting, where the abundance and distribution of preferred and non-preferred codons along mRNAs would play an important role, especially under conditions requiring rapid protein synthesis in yeast.

## Data availability statement

The datasets generated and analyzed during the current study are available at: https://doi.org/10.34691/UCHILE/9OAUT6. The raw data statistics are available in the NCBI repository, BioProject accession number PRJNA966916.

## Author contributions

MB: Conceptualization, Formal analysis, Funding acquisition, Investigation, Methodology, Project administration, Writing – original draft, Writing – review & editing. DS: Investigation, Methodology, Validation, Writing – review & editing. VC: Formal analysis, Writing – review & editing, Conceptualization. JA: Conceptualization, Formal analysis, Writing – original draft, Writing – review & editing.

## Supplementary material

Supplementary Figure 1 Distribution of preferred codons percentages in ORFs classified by cellular pathways in all yeasts. The full names of the yeast species are given in the Supplementary Table 3.

Supplementary Table 1 Growth rates (h-1) of *P. rhodozyma* isolates.

Supplementary Table 2 Yeast culture conditions, and method for RNAseq and raw data statistics.

Supplementary Table 3 Yeast name abbreviations, optimal temperature for growth (OTG), and SRA files of yeasts studied in this work.

Supplementary Table 4 Relative synonymous codon usage (RSCU) in the yeasts studied in this work.
