# Supplementary figures and images for: Codon usage bias in yeasts and its correlation with gene expression, growth temperature, and protein structure
Source: Front Microbiol. 2024 Jul 8;15:1414422. doi: 10.3389/fmicb.2024.1414422 (PMC11260810; doi:10.3389/fmicb.2024.1414422)

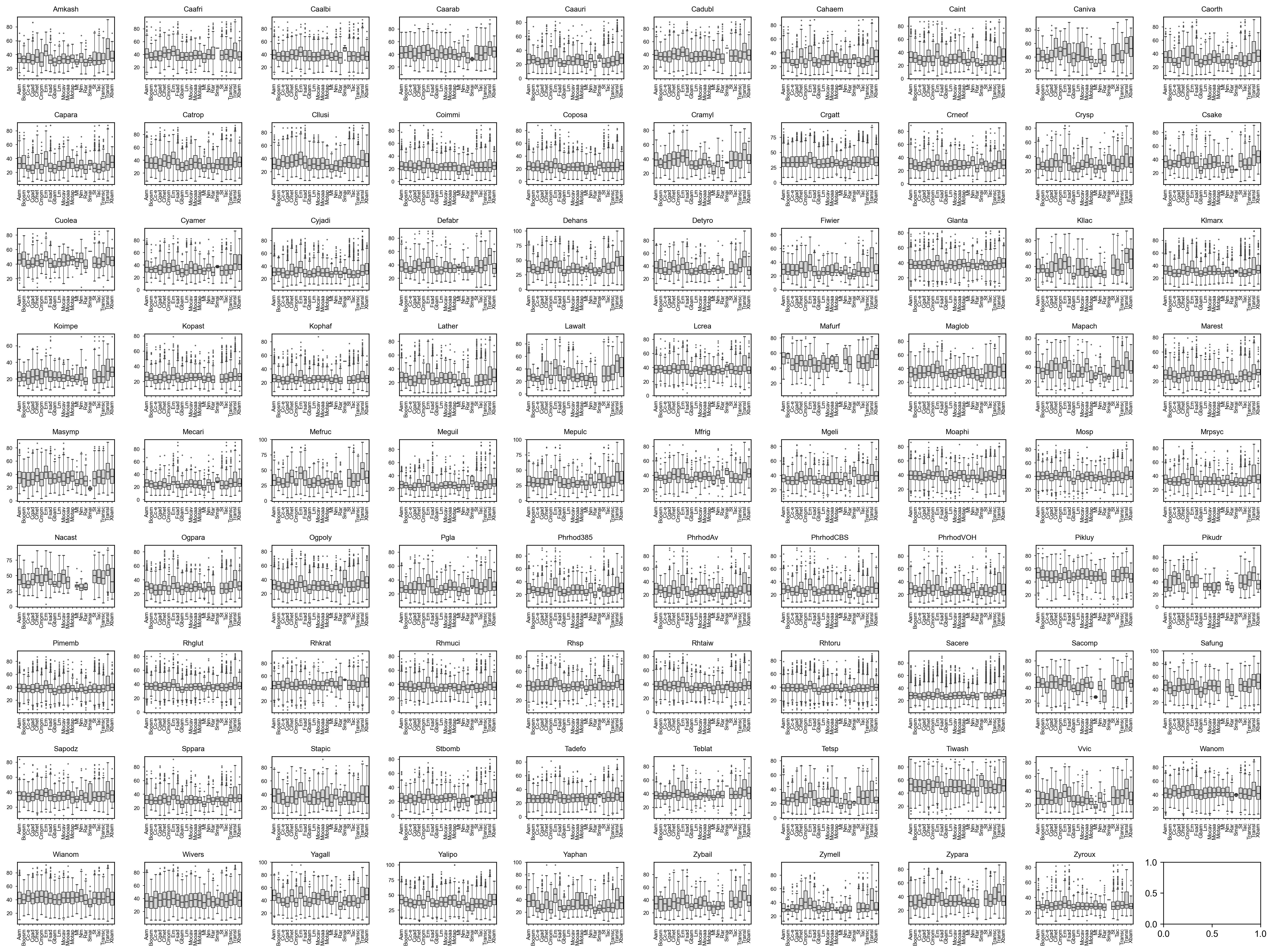

Supplement: Supplementary Figure 1 — Distribution of preferred codons percentages in ORFs classified by cellular pathways in all yeasts. The full names of the yeast species are given in the Supplementary Table 3. [file Image_1.JPEG]
